# Exercise increases MEF2A abundance in rat cardiac muscle by downregulating microRNA-223-5p

**DOI:** 10.1038/s41598-023-41696-z

**Published:** 2023-09-02

**Authors:** Elba D. Carrillo, Dulce I. Hernández, Maikel Valle Clara, Ivonne Lezama, María C. García, Jorge A. Sánchez

**Affiliations:** Departamento de Farmacología, Centro de Investigación y de Estudios Avanzados del IPN, Av. Instituto Politécnico Nacional 2508, CP 07360 Mexico City, Mexico

**Keywords:** Biochemistry, Physiology

## Abstract

Exercise plays an important role in cardiac health and enhances the transport of glucose in cardiac muscle by increasing the glucose transporter-4 (GLUT4) content at the cell membrane. The GLUT4 gene is a target of myocyte enhancer transcription factor 2A (MEF2A). Several transcription factors are regulated by microRNAs (miRs), small non-coding RNAs that control gene expression at the posttranscriptional level. In this study we tested the hypothesis that exercise regulates the expression of miR-223 and that MEF2A is a direct target of miR-223. Quantitative reverse transcriptase polymerase chain reaction (qRT-PCR) and western blot experiments showed that GLUT4 gene expression and protein abundance increased by 30 and 23%, respectively, in the microsomal fraction immediately after exercise, and had returned to control levels after 18 h. In contrast, the increase in GLUT4 in the membrane fraction was delayed. Exercise also increased the protein abundance of transcription factors involved in GLUT4 expression. Immediately after exercise, the protein abundance of MEF2A, nuclear respiratory factor 1 (NRF1), and forkhead box O1 (FOXO1) increased by 18, 30, and 40%, respectively. qRT-PCR experiments showed that miR-223-3p and miR-223-5p expression decreased immediately after exercise by 60 and 30%, respectively, and luciferase assays indicated that MEF2A is a target of the 5p strand of miR-223. Overexpression of miR-223-5p in H9c2 cells decreased the protein abundance of MEF2A. Our results suggest that the exercise-induced increase in GLUT4 content in cardiac muscle is partly due to the posttranscriptional increase in MEF2A protein abundance caused by the decrease in miR-223-5p expression. The exercise-induced decrease in miR-223-3p expression likely contributes to the increases in NRF1 and FOXO1 abundance and GLUT4 content.

## Introduction

The rhythmic contractile activity of the heart has large energy requirements. To meet this demand, the heart uses a variety of energy sources, such as glucose, glycogen, lactate, pyruvate, various amino acids, long-chain fatty acids, and triacyl-glycerols. Under resting conditions, the heart’s major energy source is the mitochondrial β-oxidation of fatty acids^1^.

Exercise is generally accepted to have beneficial effects on the heart in health and disease, and the characterization of the mechanisms involved is thus of obvious importance. Exercise leads to cardiac adaptations and changes in the energetic substrates required to sustain cardiac contractility. Specifically, myocardial glucose uptake increases with exercise^2,3^. Glucose transporter-4 (GLUT4) is the predominant glucose transporter in the adult heart^4^, and it is translocated to the plasma membrane, thereby facilitating glucose uptake, during high-intensity exercise^5^. The GLUT4 gene is a direct target of myocyte enhancer factor 2 (MEF2), a transcription factor that plays a central role in the transmission of extracellular signals to the genome and forms a specific DNA–protein complex with the promoter regulatory region of GLUT4 in heart and other tissues^6^. Among the several isoforms of MEF2, MEF2A is selectively down-regulated in insulin-deficient diabetes^7^. Swimming exercise increases the expression of cardiac GLUT4 mRNA^8^ and the binding of MEF2A to the skeletal muscle GLUT4 promoter^9^, pointing to a significant role of this transcription factor in the beneficial metabolic effects of exercise. Moreover, the Forkhead box (FOX) subfamily of transcription factors that includes FOXO1, FOXO3, and FOXO4 is critical for the maintenance of cardiac function in adults and is involved in the regulation of metabolism^10^. An experiment involving cardiomyocyte-specific FOXO1 knockout showed that FOXO1 is required for exercise-induced cardiac hypertrophy^11^.

In heart and other tissues, exercise also regulates the expression of microRNAs (miRs)^12^, which are small non-coding RNAs involved in the posttranscriptional regulation of gene expression, mainly via base pairing with the 3´or 5´-untranslated regions (UTRs) of target mRNAs^13^. Although the preservation of one strand of a miR and degradation of the other is commonly observed^14^, both strands are preserved in several cases^15^, including in the heart, where miR-223-3p and miR-223-5p are significantly expressed^16–18^. miR-223-3p targets FOXO1^19^. miR-133 and miR-1 are examples of miRs that are down-regulated by exercise. They are involved in the promotion of pathological hypertrophy, and their down-regulation improves cardiac function in rats^12^. miR-133 indirectly regulates the expression of GLUT4 by targeting other mRNAs in cardiomyocytes, such as that of Krüppel-like factor 15^20^. On the other hand, based on experiments in which miR-223 in cardiomyocytes has been overexpressed by adenovirus infection, it has been proposed that this miR directly regulates GLUT4^21^. However, the possibility that exercise regulates the expression of GLUT4 through miRs by targeting MEF2A has remained largely unexplored.

In this study, we used a rat swimming protocol to test the hypothesis that exercise increases the protein abundance of MEF2A in rat cardiac muscle, with corresponding increases in GLUT4 gene expression and protein abundance. MEF2A is a target of transcription factor NRF1^22^, therefore we tested the hypothesis that NRF1 is also upregulated by exercise. miRs are well known repressors of protein translation^13^. Therefore, we speculated that a decrease in the expression of specific miRs might be involved in the increase in protein abundance in MEF2A and consequently in that of GLUT4. To test this possibility, we measured the expression of miR-223 and miR-133 after exercise and tested whether miR-223 binds to the 3´UTR region of MEF2A.

## Results

### Exercise increases GLUT4 mRNA expression and protein abundance and MEF2A and NRF1 abundance

**Figure 1:**
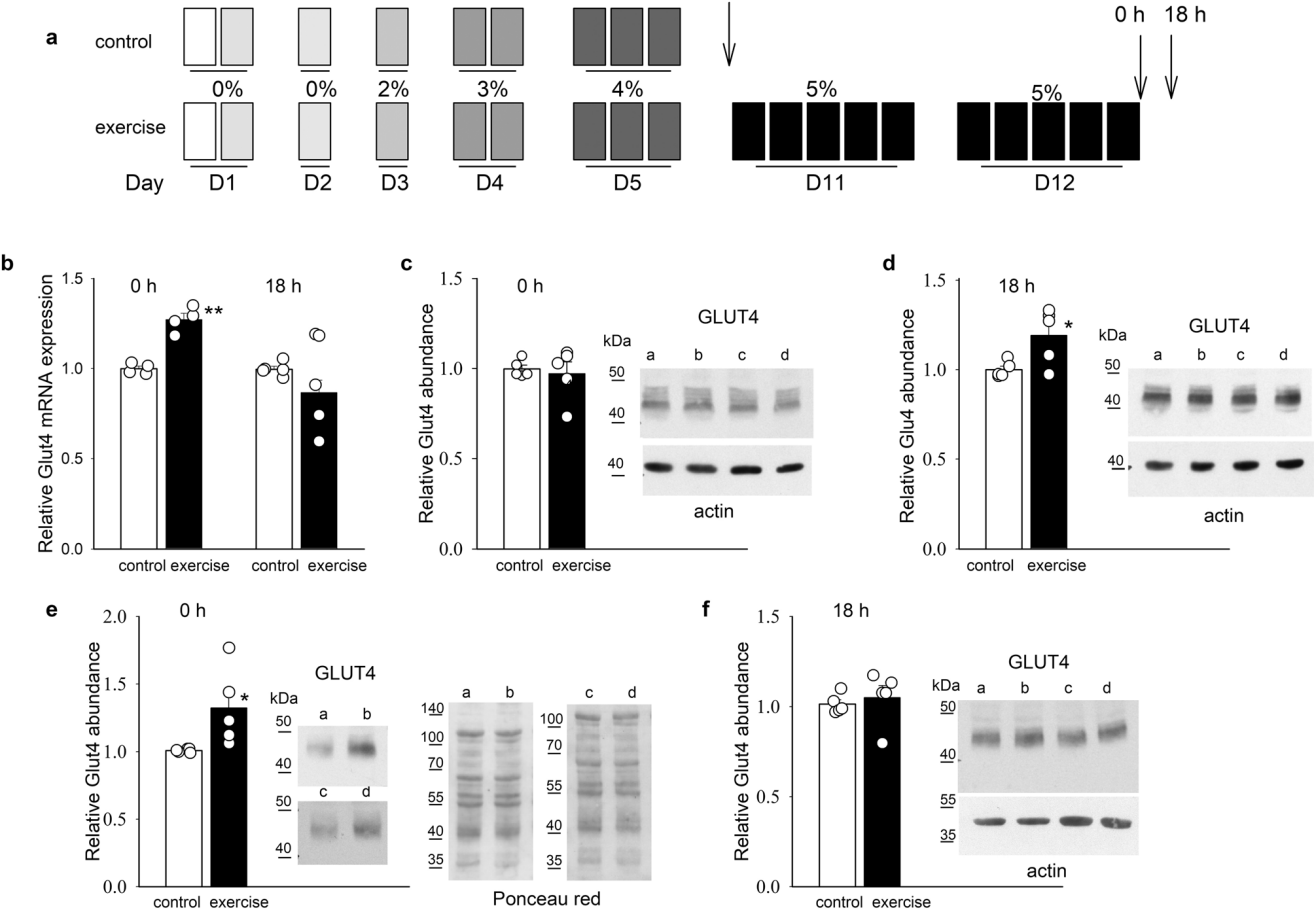
Effects of exercise on GLUT4 mRNA expression and protein abundance. (**a**) Swimming protocol. The weight attached to the tails of the exercising rats is expressed as a percentage of body weight. Arrows indicate the timing of sacrifice. (**b**) Relative GLUT4 mRNA expression in rat ventricles after exercise. (**c**–**d**) Relative GLUT4 protein abundance in the membrane fraction of rat ventricles after exercise. (**e–f**) the corresponding values from the microsomal fraction. Representative blots of GLUT4 from two separate experiments are illustrated in the insets of panels (**c**–**f**). Letters a and c indicate control conditions, and b and d, after exercise. Actin bands were used to normalize GLUT4 density values in panels (**c**), (**d**) and (**f**) and Ponceau red was used in panel (**e**). Original blots and Ponceau images are presented in Supplementary Information Fig. 1. The bar graphs represent means ± SEMs. Each symbol represents a separate experiment. *p < 0.5, ** *p* < .01.

We found increased levels of GLUT4 mRNA in the ventricles of rats sacrificed immediately after completing the exercise protocol, suggesting that exercise regulates GLUT4 expression at the transcription level. The increase was transient and had subsided completely after 18 h (Fig. 1b). Western blotting revealed a small, but significant, increase in GLUT4 protein abundance in the membrane fraction at 18 h after exercise, but no increase immediately after exercise (0 h). The density of actin bands remained unaffected and was used for normalization (Fig. 1c,d). In the microsomal fraction, GLUT4 protein abundance was increased immediately after exercise and had returned to control values after 18 h (Fig. 1e,f). Ponceau red and actin bands were used for normalization in Fig. 1e,f, respectively.

**Figure 2:**
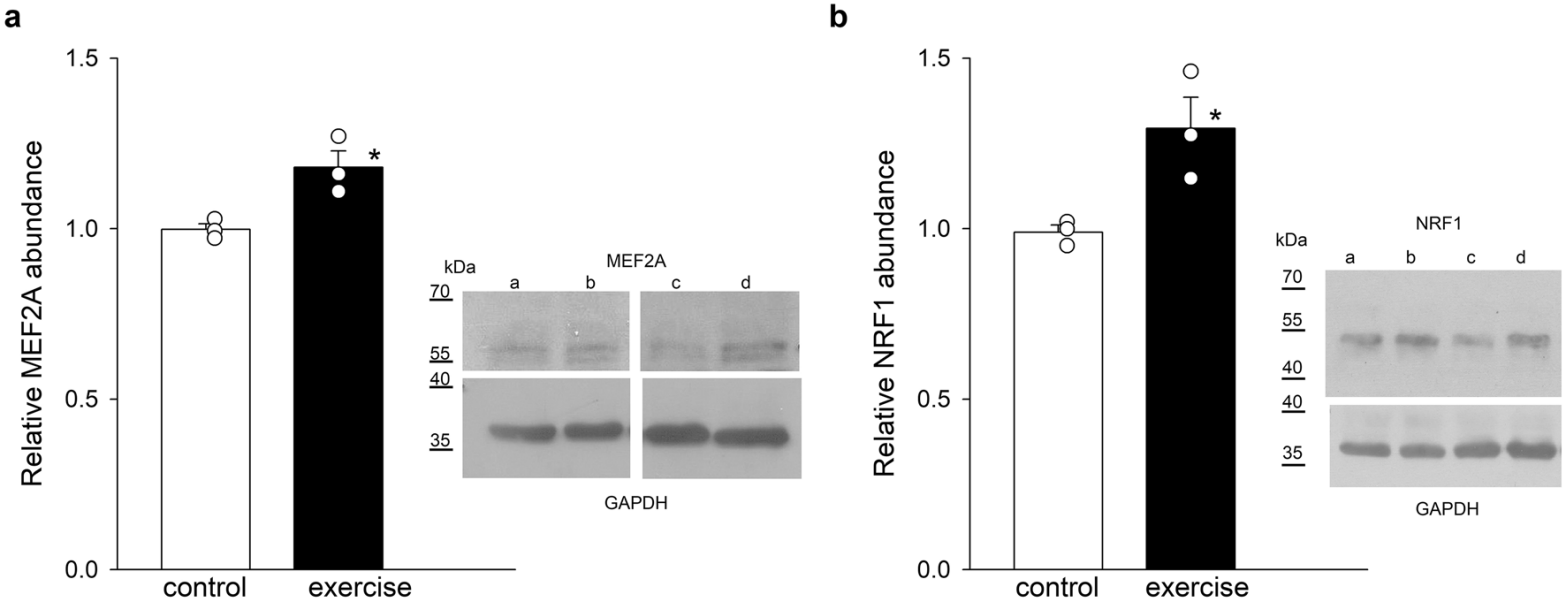
Relative abundance of MEF2A and NRF1 (**a** and **b**) in rat ventricles immediately after exercise. GAPDH density values were used for normalization. The bar graphs show means ± SEMs. Each symbol represents a separate experiment. Insets show representative blots under control conditions (letters a and c) and after exercise (letters b and d) from two separate experiments. Original blots are presented in Supplementary Information Fig. 2. *p < 0.05.

Next, we investigated whether MEF2A and NRF1 are also upregulated by exercise. Western blotting showed increases in MEF2A and NRF1 protein abundance immediately after exercise (Fig. 2a,b). The densities of GAPDH bands were unaffected and were used for normalization.

### Exercise down-regulates miR-133a-3p, miR-133b-3p, miR-223-3p, and miR-223-5p expression

**Figure 3:**
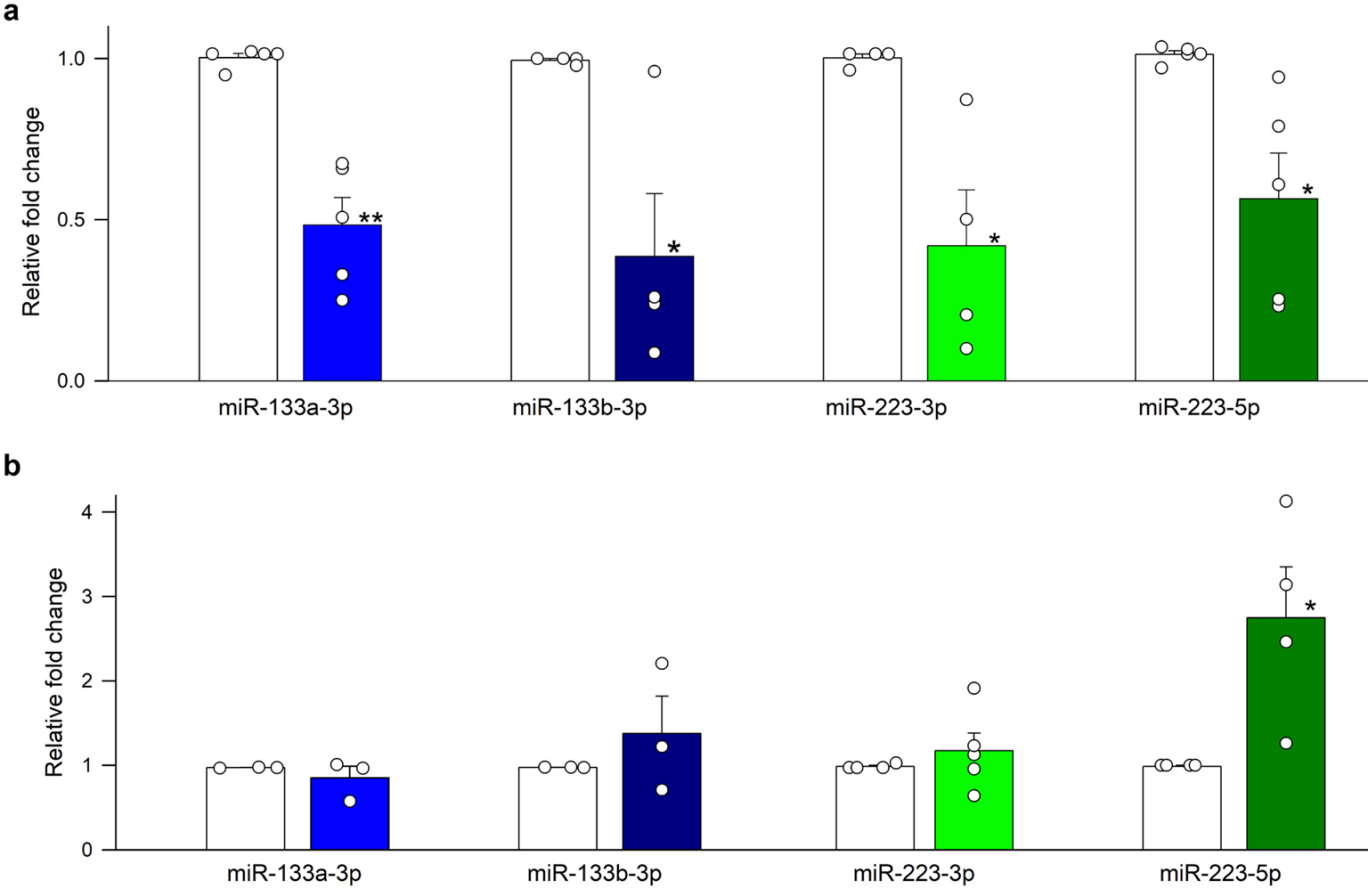
Relative miR expression in rat ventricles immediately after exercise (**a**) and after 18 h (**b**). The bar graphs represent means ± SEMs. Each symbol represents a separate experiment. *p < 0.05; **p < 0.01.

We found that exercise produced an immediate decrease in the expression of miR-223-3p, miR-223-5p, miR-133a-3p and miR-133b-3p (Fig. 3a). The expression of these miRs returned to control levels after 18 h, except miR-223-5p, which reached higher expression levels (Fig. 3b).

**Figure 4:**
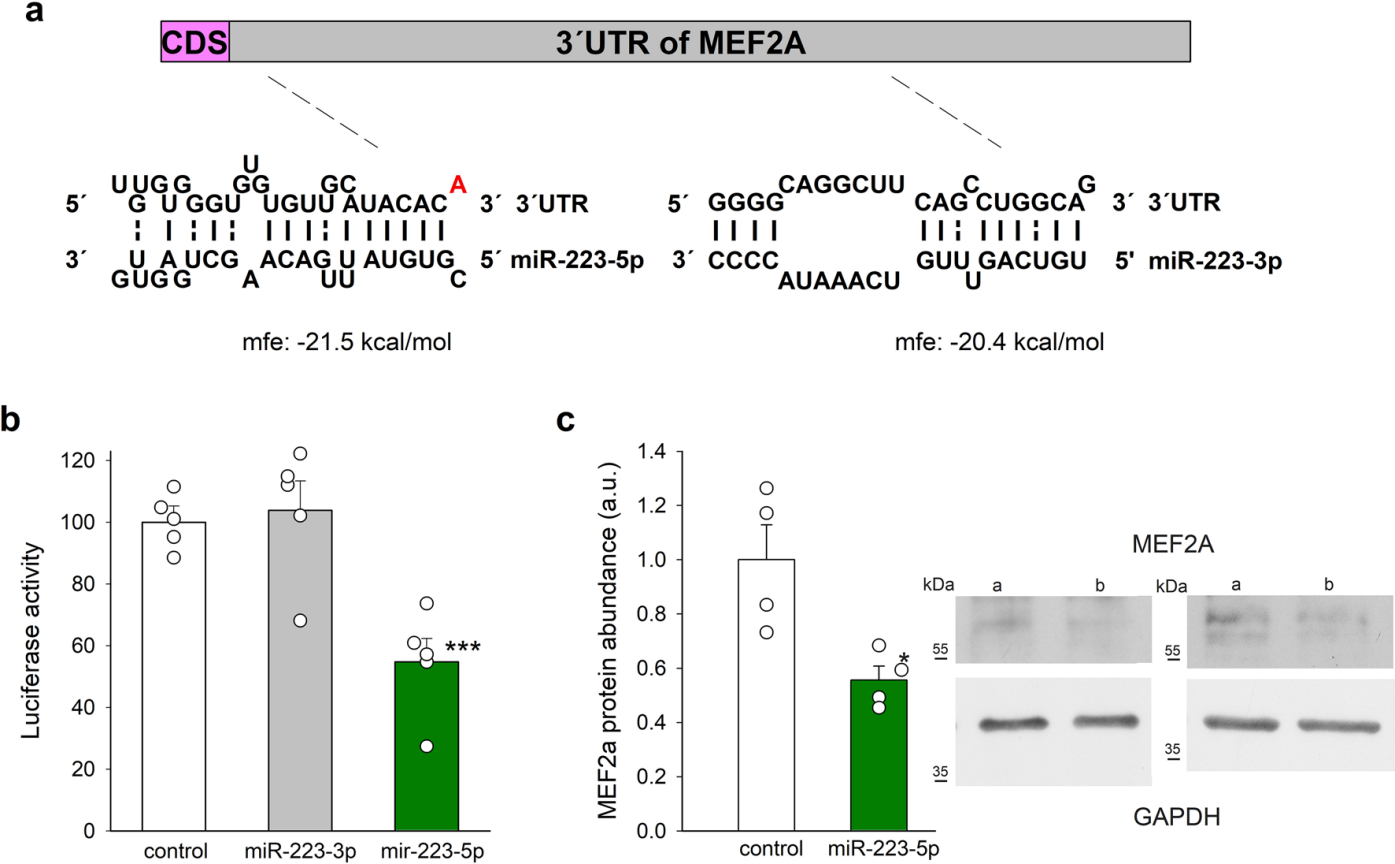
Inhibition of luciferase activity in the 3´ UTR of MEF2A by miR-223-5p. (**a**) miR-223-5p potentially binds the 3´ UTR of MEF2A with a free energy of -21.5 kcal/mol. The detailed alignment showing the position of the seed region in the 3´UTR of MEF2A-5p mRNA is shown. No such alignment was found for MEF2A-3p. Bases paired by Watson–Crick bonds are indicated by solid vertical lines, and G:U pairs are indicated by dashed lines. CDS, coding sequence. (**b**) Results of luciferase reporter assays performed with HEK293 cells. (**c**) Effects of miR-223-5p overexpression on MEF2A protein abundance in H9c2 cells. Representative blots of MEF2A and actin from two separate experiments are illustrated in the inset. Original blots are presented in Supplemenary Information Fig. 4. Data are means ± SEM, each symbol represents a separate experiment. *p < 0.05; ***p < 0.001.

In exploring whether miR-223-3p and miR-223-5p formed duplexes with the 3´ UTR of MEF2A mRNA, we found one exact seed match to positions 2–7 of miR-223-5p followed by an “A”, but none for miR-223-3p (Fig. 4a). Positions 1–6 of miR-223-3p potentially bind to the 3´ UTR region of MEF2A but this interaction is not expected to be functional (Fig. 4a). Correspondingly, miR-223-5p, but not miR-223-3p, inhibited luciferase activity in vitro (Fig. 4b). To investigate whether miR-223-5p represses MEF2A at the protein level in cardiac cells, we performed overexpression experiments on H9c2 cells, an embryonic rat heart-derived cell line. Cells were transfected with pre miR-223-5p and MEF2A abundance was measured. In agreement with Luciferase results, MEF2A protein abundance decreased (Fig. 4c**)**.

### FOXO1 is regulated by exercise

**Figure 5:**
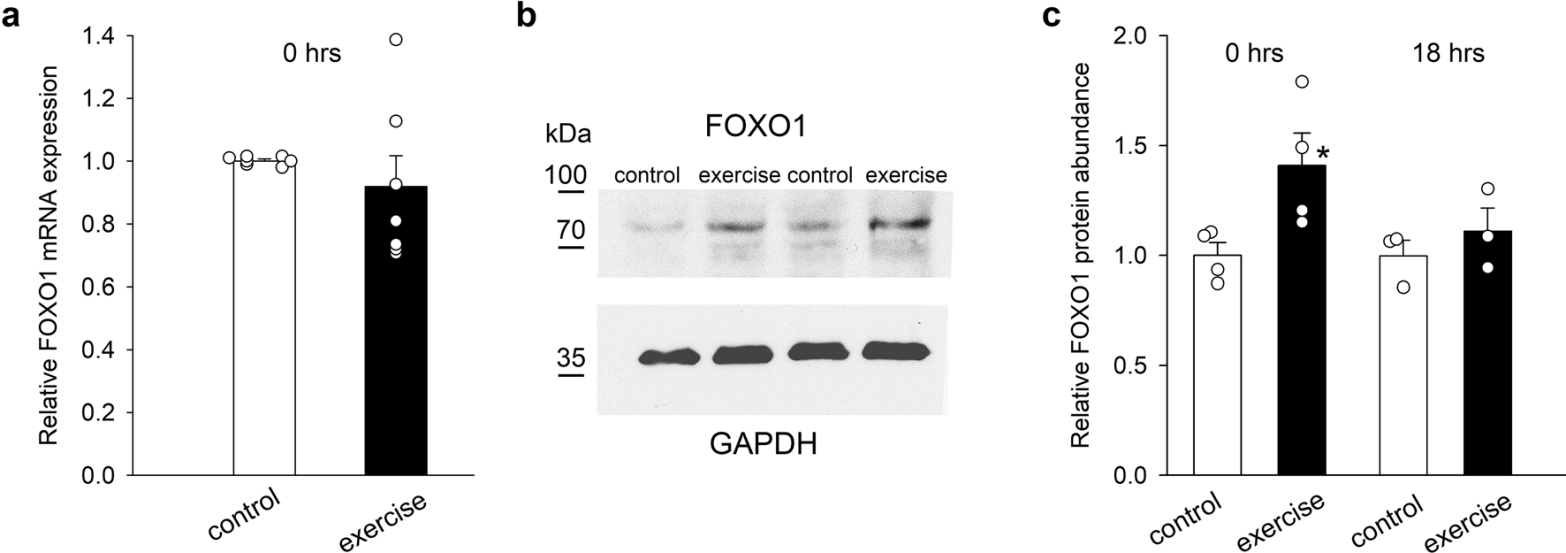
Effects of exercise on relative FOXO1 mRNA expression (**a**) and protein abundance (**b**, **c**) in rat ventricles after exercise. Representative blots of FOXO1 and GAPDH from two separate experiments are shown (**b**). Original blots are presented in Supplementary Information Fig. 5. Data are means ± SEMs. Each symbol represents a separate experiment. *p < 0.05.

We tested the hypothesis that FOXO1, a well-known target of miR-223-3p, is upregulated by exercise. FOXO1 mRNA expression showed no significant change, but the density of the FOXO1 protein band was increased immediately after exercise (Fig. 5a–c). GAPDH protein bands remained unaffected and were used for normalization (Fig. 5b). Consistent with the miR-223 results, exercise produced an increase in the FOXO1 protein abundance immediately after exercise, followed by a decline to control levels at 18 h (Fig. 5c).

## Discussion

In this study, we made the novel observations that exercise decreases the expression of both strands of miR-223 in the heart and increases the abundance of MEF2A and NRF1. We further showed that miR-223-5p targets the 3´ UTR of MEF2A mRNA, suggesting that the increase in MEF2A protein abundance is due in part to the exercise-associated decrease in miR-223-5p expression.

Given the well-documented increase in GLUT4 transcription caused by MEF2A^23,24^, the upregulation of this transcription factor likely contributed to the increased GLUT4 mRNA expression that we observed immediately after exercise, accompanied by an early increase in GLUT4 protein abundance in the microsomal fraction and a later increase at the membrane level, consistent with GLUT4 translocation. These results are in agreement with the previous observation that exercise promotes GLUT4 translocation to the plasma membrane^5^. Consistent with our observations in cardiac muscle, exercise increases the MEF2A content of skeletal muscle in mice^25^ and in human skeletal muscle^26^ (but see^9^).

In addition to the posttranscriptional regulation of MEF2A by miR-223-5p in cardiac muscle observed in this study, it is likely that exercise upregulates cardiac MEF2A through other mechanisms. In skeletal muscle, swimming exercise increases NRF1 and MEF2A gene expression^27^ and in transgenic mice that have NRF1 overexpressed, the expression of muscle MEF2A and GLUT4 and the glucose transport capacity increase^28^. A similar mechanism may operate in cardiac muscle since MEF2A mRNA is induced with the forced expression of NRF1^22^. Consistent with the observations in skeletal muscle we report for the first time that exercise enhances the NRF1 abundance in cardiac muscle. These observations suggest that this transcription factor also contributes to increases in MEF2A and GLUT4 content in cardiac muscle after exercise. Other transcription factors may also play a role, as suggested by our findings on the effects of exercise on FOXO1 gene expression and protein abundance.

Exercise greatly increases FOXO1 mRNA expression in mouse skeletal muscle^29–31^, but not in cardiac muscle^32^, as we also observed in the present experiments. Despite the lack of change in FOXO1 mRNA expression, however, we found a significant increase in FOXO1 protein abundance after exercise. This observation suggests that exercise regulates FOXO1 posttranscriptionally, most likely related to the observed decrease in miR-223-3p expression. Several lines of evidence support this conclusion. First, miR-223 transfection reduced the abundance of FOXO1 in several cell lines, as determined by western blotting^33,34^. Second, FOXO1 is a direct target of miR-223-3p, as determined by luciferase assays^19,33,34^. miR-223-3p also targets MEF2C, another transcription factor associated with cardiac morphogenesis^35^ that may also be regulated by exercise in cardiac muscle.

The exercise-induced increase in FOXO1 abundance in cardiac muscle is expected to contribute to GLUT4 upregulation, which increases glucose uptake. Transfection of the dominant-negative FOXO1 construct reduces glucose uptake and GLUT4 protein abundance in skeletal muscle^36^, and experiments performed with a muscle cell line showed that GLUT4 promoter activity is regulated by FOXO1^37^.

## Conclusion

The main findings of this work are that exercise significantly reduces the expression of both strands of miR-223 in the heart and that MEF2A is a target of miR-223-5p. Additionally we showed that exercise upregulates the GLUT4-related transcription factors MEF2A, NRF1, and FOXO1. These findings suggest that exercise-induced GLUT4 upregulation may be explained in part by the decreased expression of both strands of miR-223. Although the present experiments were performed in rats, exercise plausibly induces similar changes in the human heart. Further work is required to test this possibility. Given the clinical importance of exercise’s ability to reduce the occurrence of cardiac events in middle age, a detailed understanding of the cellular mechanisms underlying the heart’s adaption to exercise is relevant to the development of cardioprotective therapies.

## Materials and methods

### Subjects and ethics

Male Wistar rats, 7 weeks old, with an initial weight of 225 ± 5 g were used in this study. They were housed four per cage in a room maintained at a temperature between 21 and 24 °C with a 12:12-h light–dark cycle and fed standard rat chow and water ad libitum. All experiments were performed in accordance with ARRIVE guidelines. The experimental protocols were approved by CICUAL (*Comité Interno para el Cuidado y Uso de Animales de Laboratorio*) of CINVESTAV-IPN (*Centro de Investigación y de Estudios Avanzados del Instituto Politécnico Nacional*), in compliance with Mexican federal law and CONACYT (*Consejo Nacional de Ciencia y Tecnología*) regulations.

### Swimming protocol

The swimming protocol was similar to that used by Smith et al. (2007)^9^. All rats were familiarized with swimming, and a load was attached to their tails and progressively increased (Fig. 1a). The water temperature was maintained at 37 °C during the swimming sessions. On the first day, the rats were familiarized with the pool for 17 min with no water and no load attached. Then, water was added to the pool and the rats were allowed to swim for an additional 17 min. On days 2–5, the rats were subjected to swimming bouts lasting 17 min, with the number of bouts increased progressively from one to three and the load increased from 0 to 4% body weight. They were given a 3-min rest period between bouts. The rats were then allowed to rest for 6 days to eliminate any adaptation that may have resulted from the familiarization training. The rats were observed carefully during the familiarization sessions, and those that were more active were selected for two additional days (11 and 12) of swimming for five bouts with a 5%-body-weight load. After these sessions, half of the rats were immediately anesthetized with intraperitoneal injections of 50 mg kg^−1^ pentobarbital sodium and their hearts (1.5–1.7 g) were rapidly excised; the other half was allowed to rest for 18 h before sacrifice. The less-active rats were used as controls and were sacrificed at the beginning of day 11 with no additional swimming session (Fig. 1a). Fragments of the ventricles of the excised hearts were immediately submerged in RNAlater (Qiagen, Germantown, MD, USA) and stored at − 70 °C for examination by quantitative reverse-transcriptase polymerase chain reaction (qRT-PCR); other fragments were stored in liquid nitrogen until analysis by western blotting.

### Cell culture and transfection

Embryonic rat heart-derived H9c2 cells (American Type Culture Collection) were maintained in Dulbecco’s Modified Eagle’s Medium (DMEM; Gibco) supplemented with 10% fetal bovine serum (FBS; Gibco), sodium bicarbonate (1.5 g/l), penicillin (50 IU), and streptomycin (50 µg/ml) at 37 °C and 5% CO_2_ and 95% air in a humidified incubator. Cells were used at 80–90% confluence. Transfection was performed with Ambion™ Pre-miR™ precursor (AM17100), pre-miR-223-5p (PM14755), or pre-miR negative control 2 (AM17111) at a concentration of 50–100 nM, all from ThermoFisher (Waltham, MA, USA). They were introduced into H9c2 cells using Lipofectamine 2000 (Invitrogen, Carlsbad, CA, USA) according to the manufacturer's instructions. Cells were harvested 48–72 h after transfection. Lysates were used for Western blotting to evaluate MEF2A abundance.

### Western blotting

The abundance of GLUT4 in the samples’ membrane and microsomal fractions was analyzed by western blotting. The heart ventricle fragments that were stored in liquid nitrogen were pulverized with a mortar and then homogenized with a lysis buffer containing 10 mM TRIS HCl, 1 mM EDTA, and 250 mM sucrose (pH 7.4) supplemented with a protease inhibitor cocktail (Halt™; Thermo Scientific, Waltham, MA, USA), followed by centrifugation at 800×*g* for 10 min at 4 ºC. The supernatant was kept, and the pellet was resuspended in the same lysis buffer and centrifuged again at 800×*g* for 10 min at 4 ºC. Then, both supernatants were centrifuged at 31,000×*g* for 60 min at 4 ºC. The pellet containing the membrane fraction was resuspended in lysis buffer and stored in liquid nitrogen until further use. The supernatant was centrifuged at 190,000×*g* for 60 min at 4 ºC, and the pellet containing the microsomal fraction was also resuspended in lysis buffer and stored in liquid nitrogen.

The abundance of MEF2A, NRF1, and FOXO1 was assessed by western blotting of whole extracts from heart ventricles. The ventricles were pulverized and then homogenized in a buffer containing 50 mM TRIS HCl, 150 mM NaCl, 10 mM NaF, 1 mM Na_3_VO_3_, and 0.5% NP-40 (pH 7.4) supplemented with a protease inhibitor cocktail (Halt™; Thermo Scientific, Waltham, MA, USA). The lysates were maintained on ice for 1 h and vortexed every 10 min. The samples were then centrifuged at 16,000×*g* for 15 min at 4 °C, and the supernatant was stored in liquid nitrogen until further use. The protein abundance of MEF2A from H9c2 cells was assessed following a similar protocol.

For western blotting, protein contents were measured with a Bradford protein assay^38^ and 45–60 μg of each fraction was subjected to 11% sodium dodecyl sulfate polyacrylamide gel electrophoresis (140 V, 120 min). The resultant protein bands were transferred onto nitrocellulose membranes, and the membranes were blocked with 4.5% nonfat dried milk in phosphate-buffered saline (PBS) and probed with anti-GLUT4 polyclonal antibody (ab654, 1:5000; Abcam, Waltham, MA, USA), anti-MEF2A polyclonal antibody (PA5-27380, 1:500; Invitrogen, Carlsbad, CA, USA), anti-NRF1 monoclonal antibody (ab175932, 1:1000; Abcam, Waltham, MA, USA), anti-FOXO1 monoclonal antibody (C29H4, 1:1000; Cell Signaling Technology, Danvers, MD, USA), anti-actin monoclonal antibody (A3853, 1:2000; Sigma-Aldrich, Burlington MA, USA), and anti-GAPDH monoclonal antibody (G8795, 1:10,000; Sigma Aldrich, Burlington MA, USA) in PBS for 12–14 h at room temperature. After rinsing with PBS–Tween 20 (0.1%), the blots were incubated for 1 h with anti-rabbit or anti-mouse horseradish peroxidase–conjugated secondary antibody (1:90,000; Invitrogen, Carlsbad, CA, USA) in PBS and then rinsed again with PBS-Tween 20 (0.1%). Antibody labeling was detected with Immobilon western reagent (Millipore, Billerica, MA, USA) according to the manufacturer’s instructions. Densitometry analysis was performed by integrating band densities as described elsewhere (Tammineni et al., 2018).

### qRT-PCR assays

Total RNA was isolated with an miRNeasy kit (Qiagen, Germantown, MD, USA). RNA was quantified using spectrophotometry (Implen I NanoPhotometer, Westlake Village, CA, USA). qRT-PCR was performed with 500 ng deoxyribonuclease-treated RNA in 20-µl reactions. Complementary DNA was synthesized using Superscript III RT (Invitrogen, Carlsbad, CA, USA) and random hexamers (250 ng) according to the manufacturer’s instructions. To quantify mRNA, we used TaqMan™ assays (catalog no. 4331182; Applied Biosystems, Foster City, CA, USA) with an iCycler iQ device (Bio-Rad, Hercules, CA, USA), and the following products from Applied Biosystems (Foster City, CA, USA): TaqMan gene expression master mix (catalogue no. 4369016), and the following primer–probe sets: Foxo1 (ID Rn01494868_m1), Slc2a4 (ID Rn00562597_m1), and eukaryotic 18S ribosomal RNA (ID Hs99999901_s1) as an internal control to normalize expression values.

### miR assays

Standard TaqMan™ miR assays (catalog no. 4427975; Applied Biosystems, Foster City, CA, USA), which employ target-specific stem-loop reverse transcription primers for 3’ extended templates, were used to determine the relative expression levels of rno-miR-133a-3p (ID 002246), rno-miR-133b-3p (ID 002247), rno-miR-223-3p (ID 000526), and rno-miR-223-5p (ID 007896-mat) with TaqMan Universal PCR master mix no AmpErase UNG (ID 4324018) and an iCycler iQ device (Bio-Rad, Hercules, CA, USA). miR expression was assessed relative to the small nucleolar RNA U87 (ID 001712, Applied Biosystems, Foster City, CA, USA), as recommended by the manufacturer. Changes in expression levels were determined using the 2^−ΔΔCT^ method^39^. TargetScan (https://www.targetscan.org/) and RNAhybrid (https://bibiserv.cebitc.uni-bielefeld.de/rnahybrid/), developed by^40^, were used to predict miR targeting to the 3´-UTR of MEF2A mRNA.

### Luciferase reporter assays

For luciferase reporter assays, HEK293 cells were seeded in 24-well plates at a density of 4 × 10^4^ cells/well and cultured for 24 h in Dulbecco modified Eagle medium supplemented with 10% fetal bovine serum at 37 °C and 5% CO_2_. The cells were transfected with 50 ng miR target clone control vector (pEZX, CmiT000001-MT05; GeneCopoeia) or miR target clone 3´-UTR Mef2a (RmiT046808-MT05; GeneCopoeia, Rockville, MD, Unites States). Co-transfection was performed with Ambion™ Pre-miR™ precursor (AM17100), pre-miR-223-3p (PM10903), pre-miR-223-5p (PM14755), or pre-miR negative control 2 (AM17111) all from ThermoFisher (Waltham, MA, USA) at a concentration of 100 nM using Lipofectamine 2000 (Invitrogen, Carlsbad, CA, USA) according to the manufacturer’s instructions. After 48 h, the transfection medium was collected and stored at − 70 °C for further analysis. The Secrete-Pair™ dual luminescence assay kit (LF032; GeneCopoeia, Rockville, MD, Unites States) was used to analyze the activities of *Gaussia* luciferase (GLuc) and secreted alkaline phosphatase (SEAP). For the comparison of GLuc activity among samples, the luciferase values were normalized relative to SEAP activity according to the manufacturer´s instructions. All reporter assays were performed in duplicate or triplicate. The luciferase assays were performed using a TD-20/20 luminometer (Turner Designs, San Jose, CA, USA).

### Data analysis

The data are expressed as means ± standard errors of the mean. The statistical analyses were performed using GraphPad Prism 4.0 (GraphPad Software) and Sigma Stat 2.0. For between-group comparisons, Student’s *t* test was performed (Supplementary material). For multiple-group comparisons of normally distributed data, one-way analysis of variance followed by Tukey’s honestly significant difference test was used. *P* values < 0.05 were considered to be significant.

### Supplementary Information

Supplementary Figures.

## Data Availability

The data and materials used and/or analyzed during the current study are available from the corresponding author on reasonable request.

## Abbreviations

GLUT4: Glucose transporter-4
MEF2A: Myocyte enhancer transcription factor 2A
MiRs: MicroRNAs
qRT-PCR: Quantitative reverse transcriptase polymerase chain reaction
NRF1: Nuclear respiratory factor 1
FOXO1: Forkhead box O1
UTRs: 3′ or 5′-untranslated regions
