# Supplementary material for: Exercise increases MEF2A abundance in rat cardiac muscle by downregulating microRNA-223-5p
Source: Sci Rep. 2023 Sep 2;13:14481. doi: 10.1038/s41598-023-41696-z (PMC10475133; doi:10.1038/s41598-023-41696-z)

Uncropped blot images for Carrillo *et al*. "**Exercise increases MEF2A abundance in rat cardiac muscle by downregulating microRNA-223-5p**"

S1

Fig 1**c**

Nitrocellulose membranes, containing the protein bands, were routinely cropped before hybridization. Membrane corners are indicated by arrows.

Lanes 1 and 3, control

Lanes 2 and 4, exercise

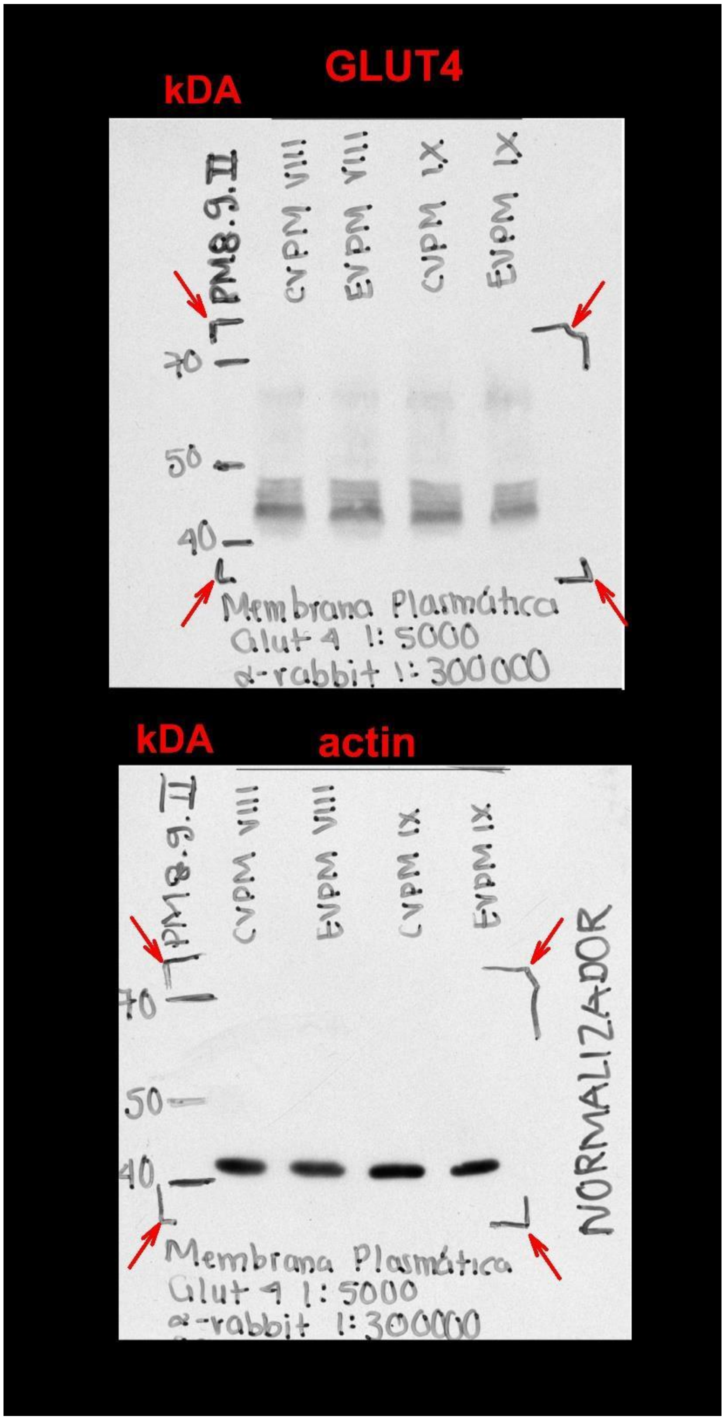

S2

Fig 1**d**

Lanes 1 and 3, control

Lanes 2 and 4, exercise

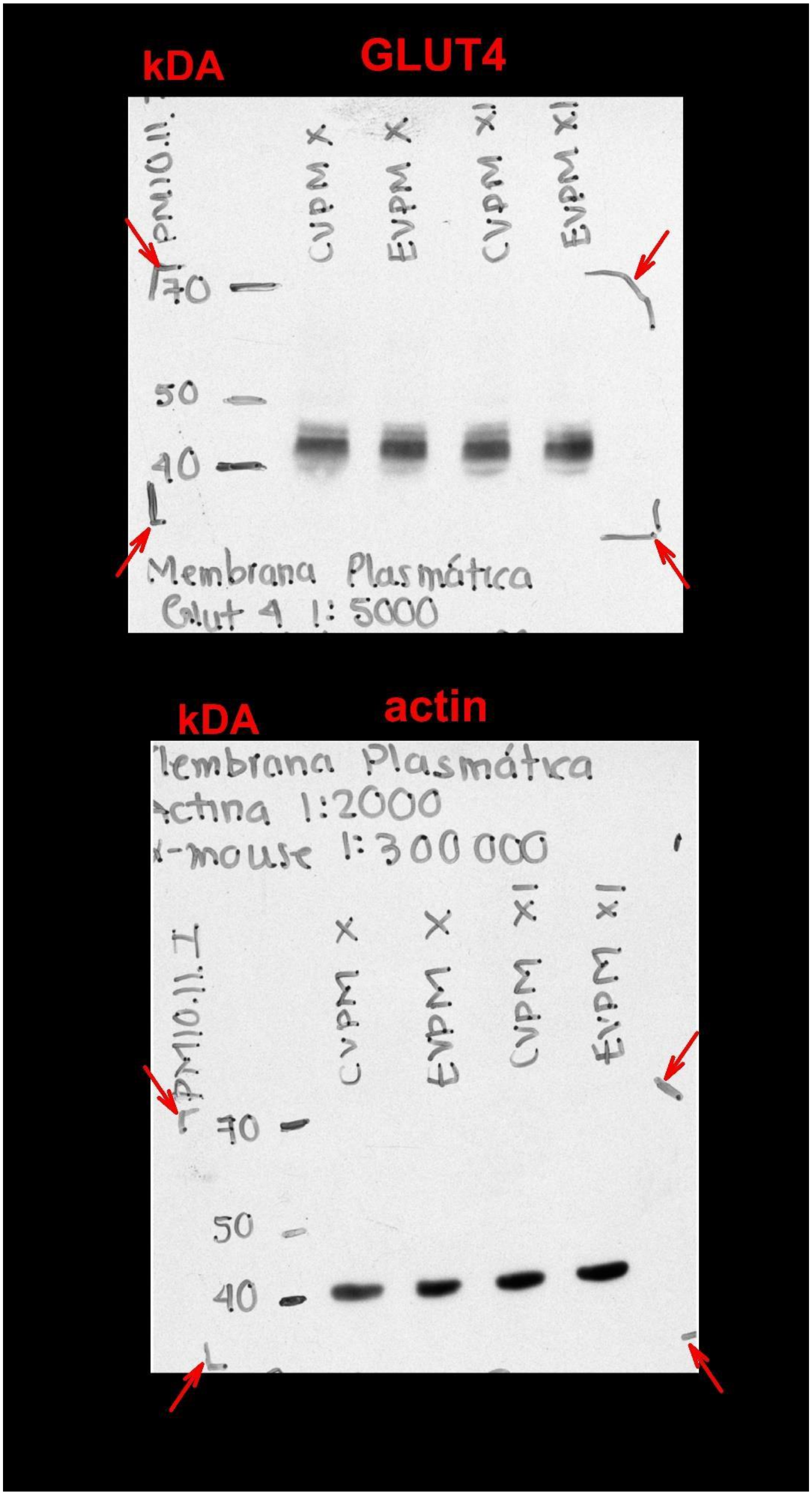

S3

Fig 1**e**, left panels

Red boxes indicate areas of blots shown in the figure.

Lanes 1 and 3, control

Lanes 2 and 4, exercise

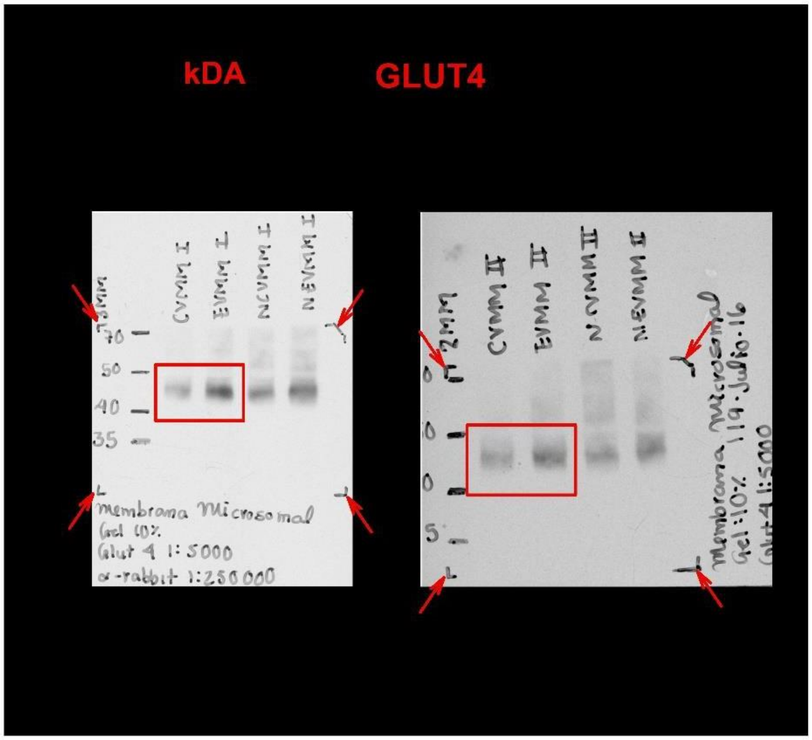

Fig 1**e**, right panels

Red boxes indicate areas of Ponceau red images shown in the figure.

Lane 1, control

Lane 2, exercise

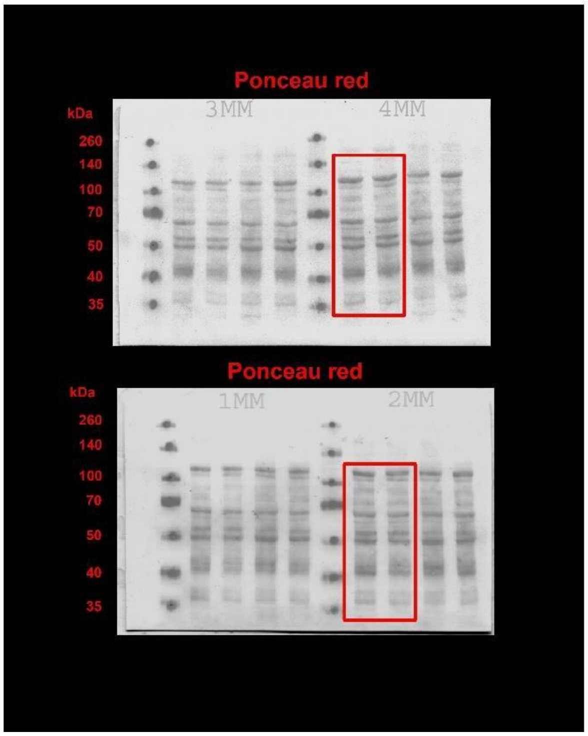

S4

Fig 1**f**

Lanes 1 and 3, control

Lanes 2 and 4, exercise

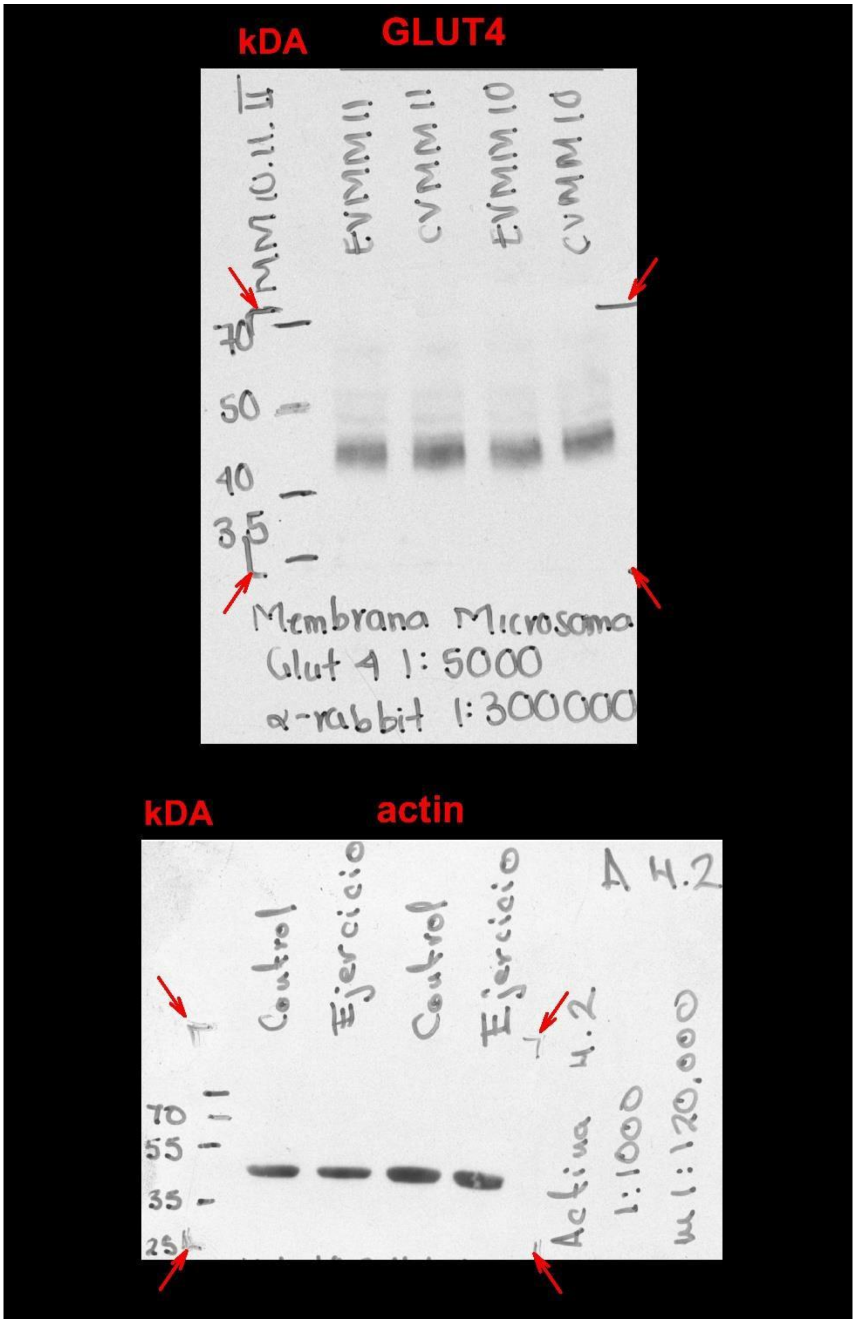

S5

Fig 2**a**

Nitrocellulose membranes, containing the protein bands, were routinely cropped before hybridization. Membrane corners are indicated by arrows.

Red boxes indicate areas of blots shown in the figure.

Lanes 1 and 5, control

Lanes 2 and 6, exercise

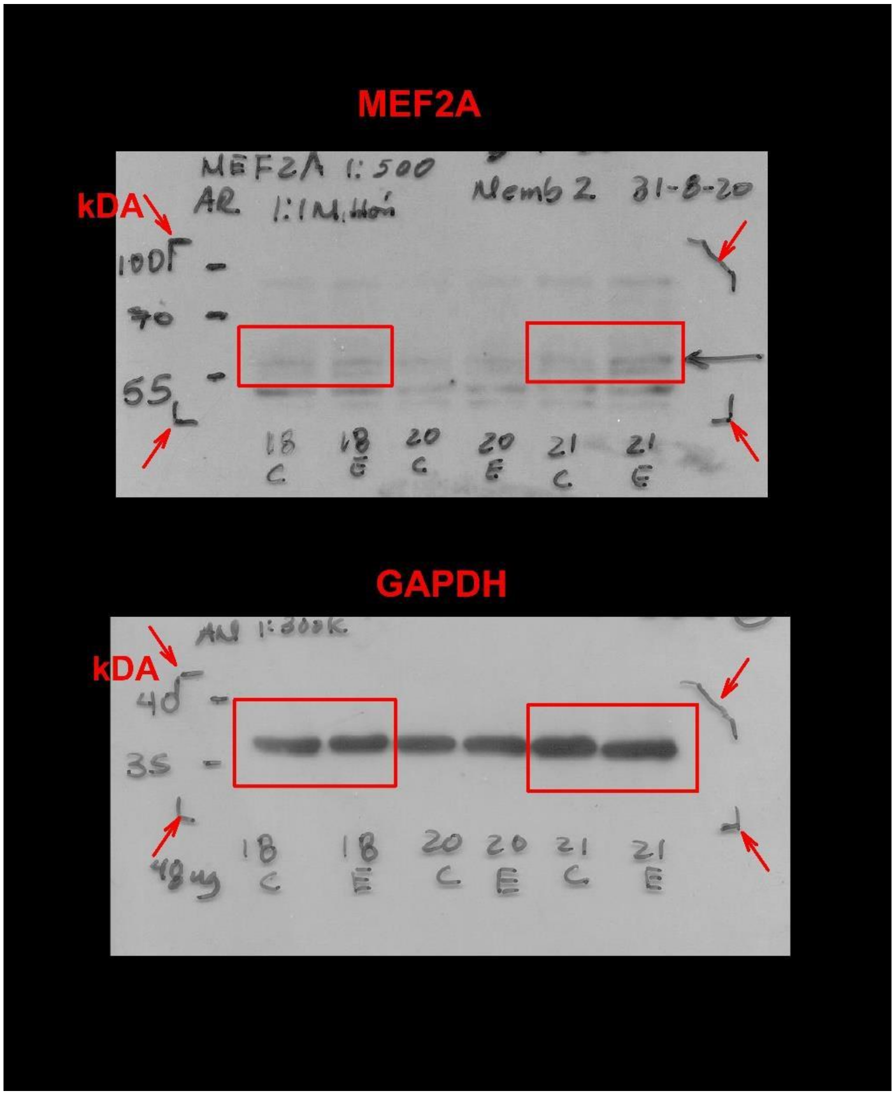

S6

Fig 2**b**

Red boxes indicate areas of blots shown in the figure.

Lanes 1 and 3, control

Lanes 2 and 4, exercise

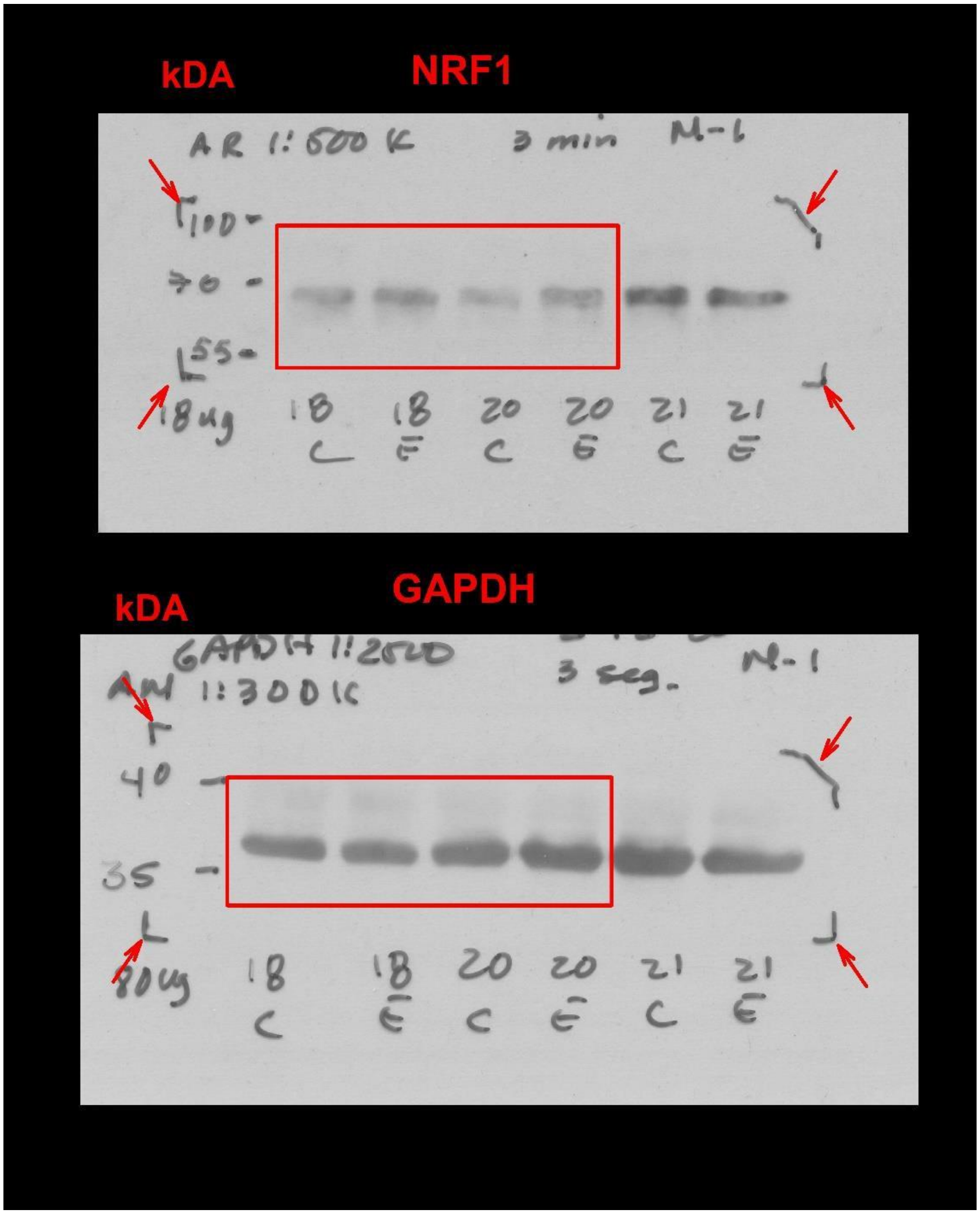

S7

Fig 4 **c**

Nitrocellulose membranes, containing the protein bands, were routinely cropped before hybridization. Membrane corners are indicated by arrows.

Red boxes indicate areas of blots shown in the figure.

Lane 5, control

Lane 6, miR-223-5p

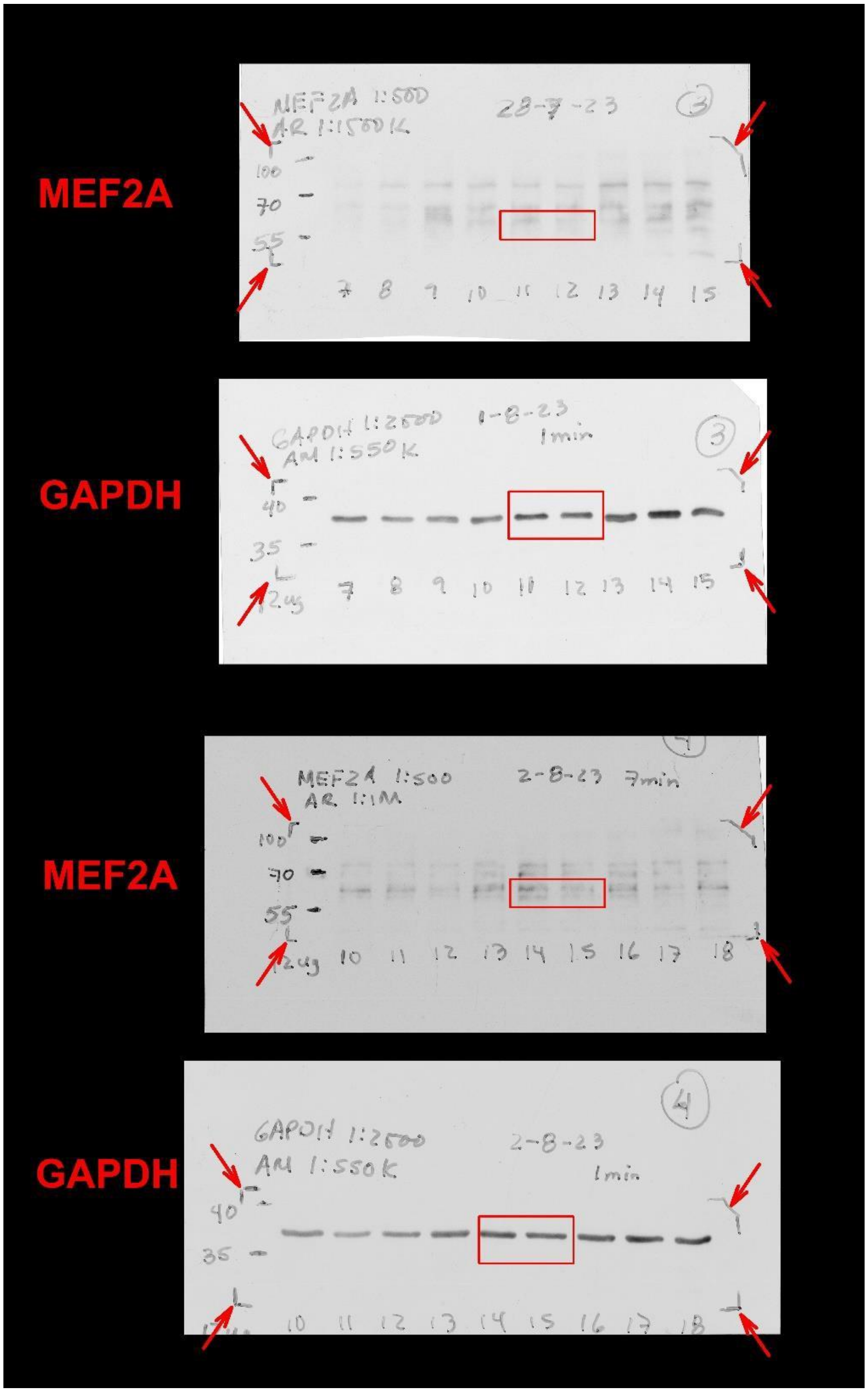

S8

Fig 5**b**

Nitrocellulose membranes, containing the protein bands, were routinely cropped before hybridization. Membrane corners are indicated by arrows.

Lanes 1 and 3, control

Lanes 2 and 4, exercise

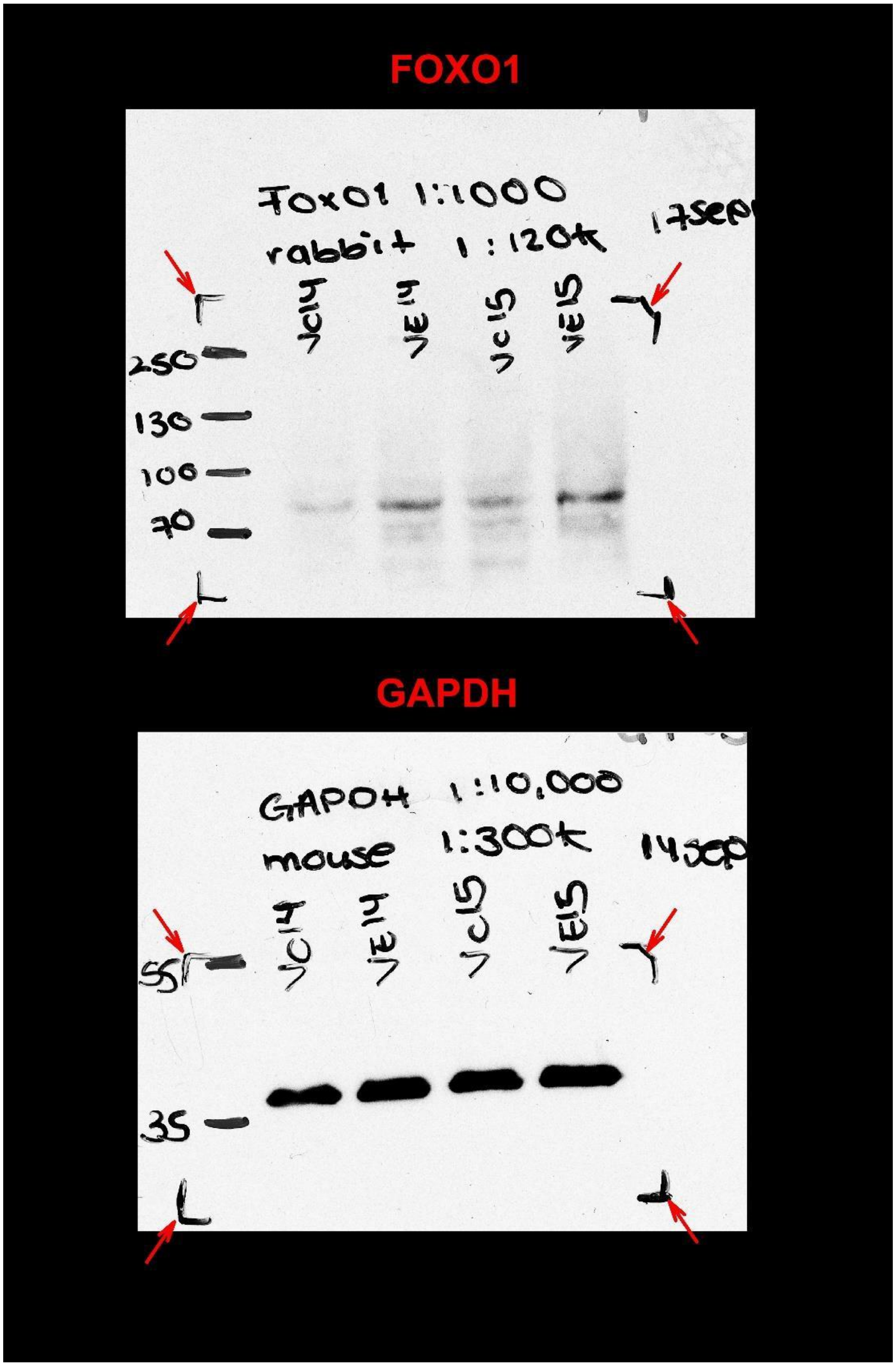

Additional blot images for Carrillo *et al*. "**Exercise increases MEF2A abundance in rat cardiac muscle by downregulating microRNA-223-5p**"

S9

Western blots of three independent experiments (**a**-**c**) showing the respective target bands of plasma membrane GLUT4 (left panels) and actin (rights panels). Left lanes, control. Right lanes, immediately after exercise (0 h). Red boxes indicate areas of blots not shown in Figure 1**c**.

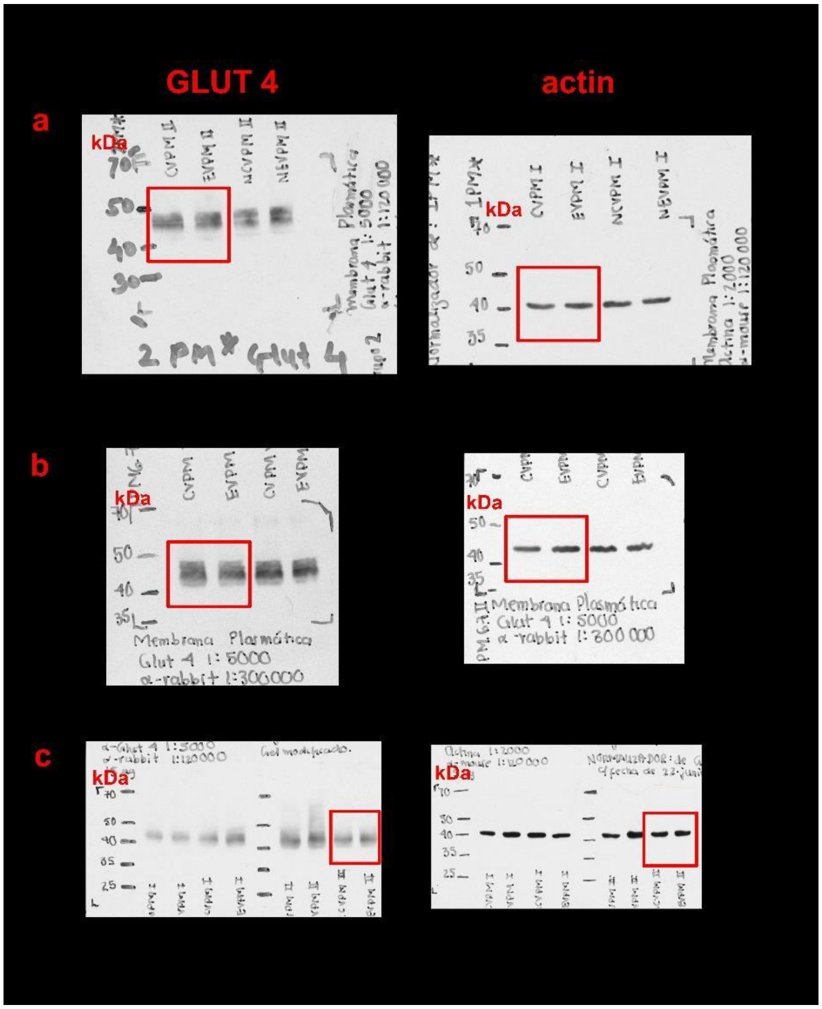

S10

Western blots of three independent experiments (**a**-**c**) showing the respective target bands of plasma membrane GLUT4 (left panels) and actin (rights panels). Left lanes, control. Right lanes, 18 h post exercise. Red boxes indicate areas of blots not shown in Figure 1**d**.

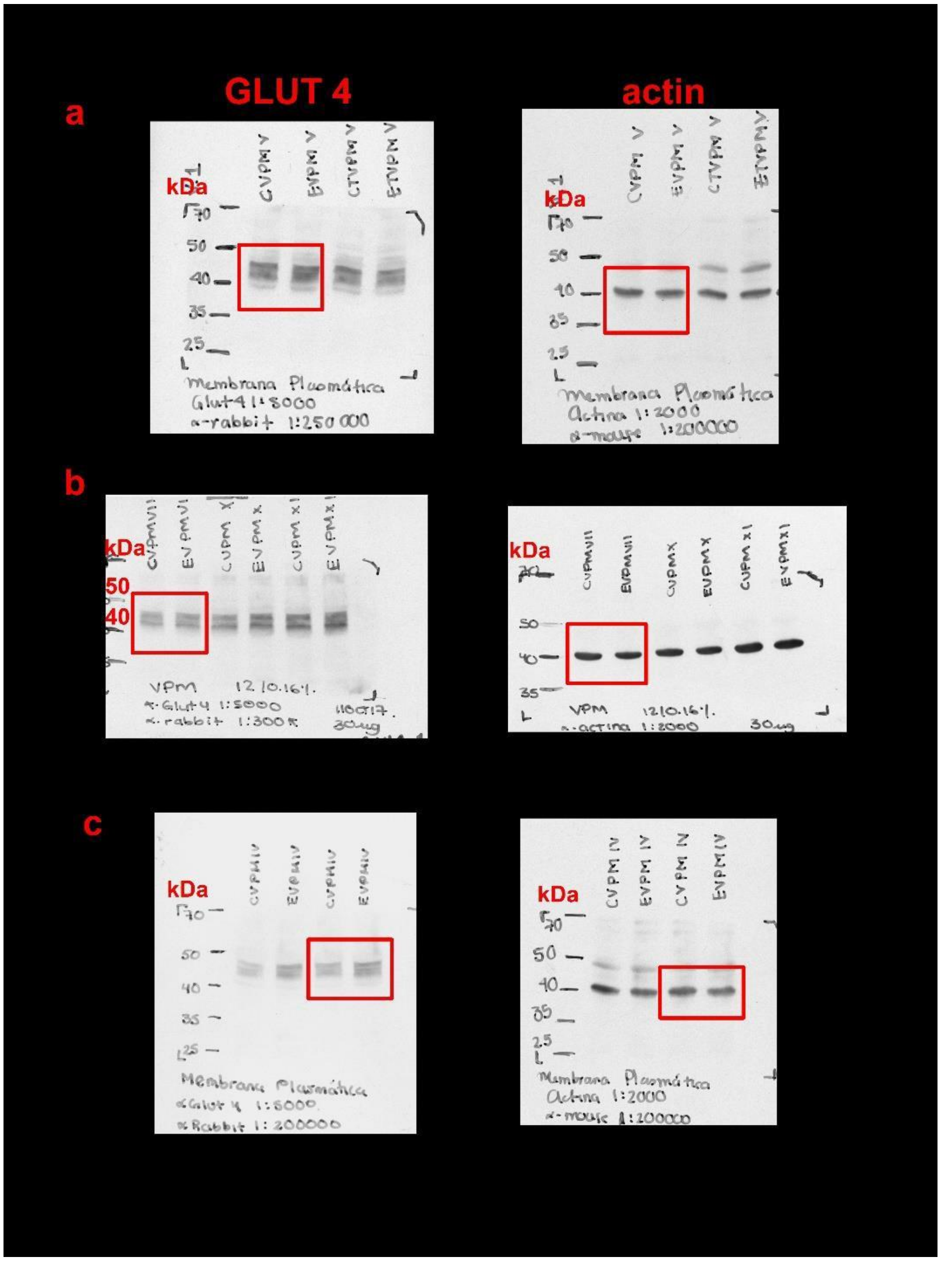

S11

Western blots of three independent experiments (**a**) showing the respective target bands of microsomal fraction GLUT4, immediately after exercise (0 h). Control and exercise bands are indicated by letters in blue.

b, the corresponding western blots from three independent experiments, 18 h post exercise. Left lanes, control and right lanes, post exercise.

Red boxes indicate areas of blots not shown in Figure 1 **e-f**.

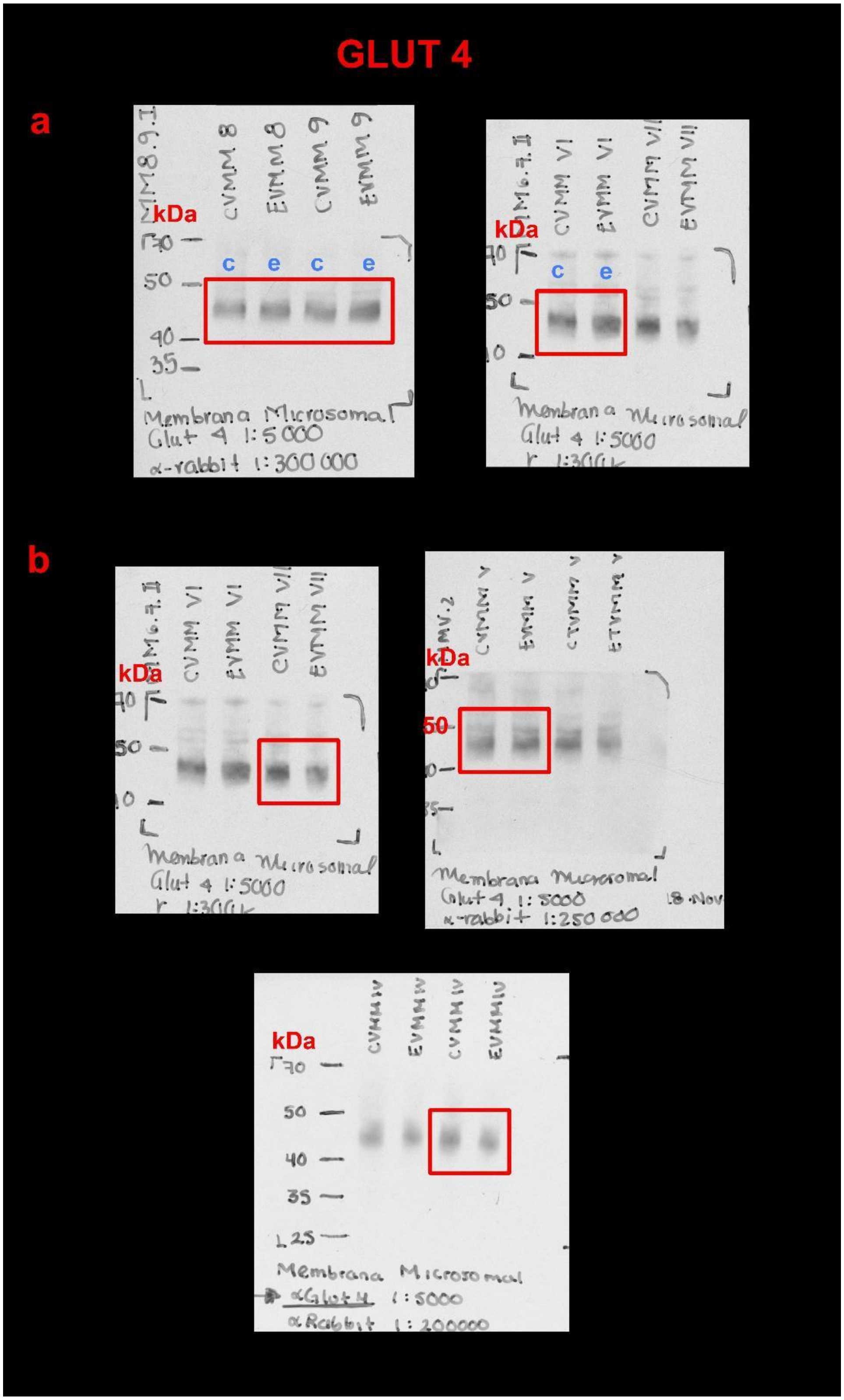

S12

Western blots showing the respective target bands of MEF2A, NRF1 and GAPDH, after exercise. Left lanes, control and right lanes, post exercise. Red boxes indicate areas of blots not shown in Figure 2 **a-b**.

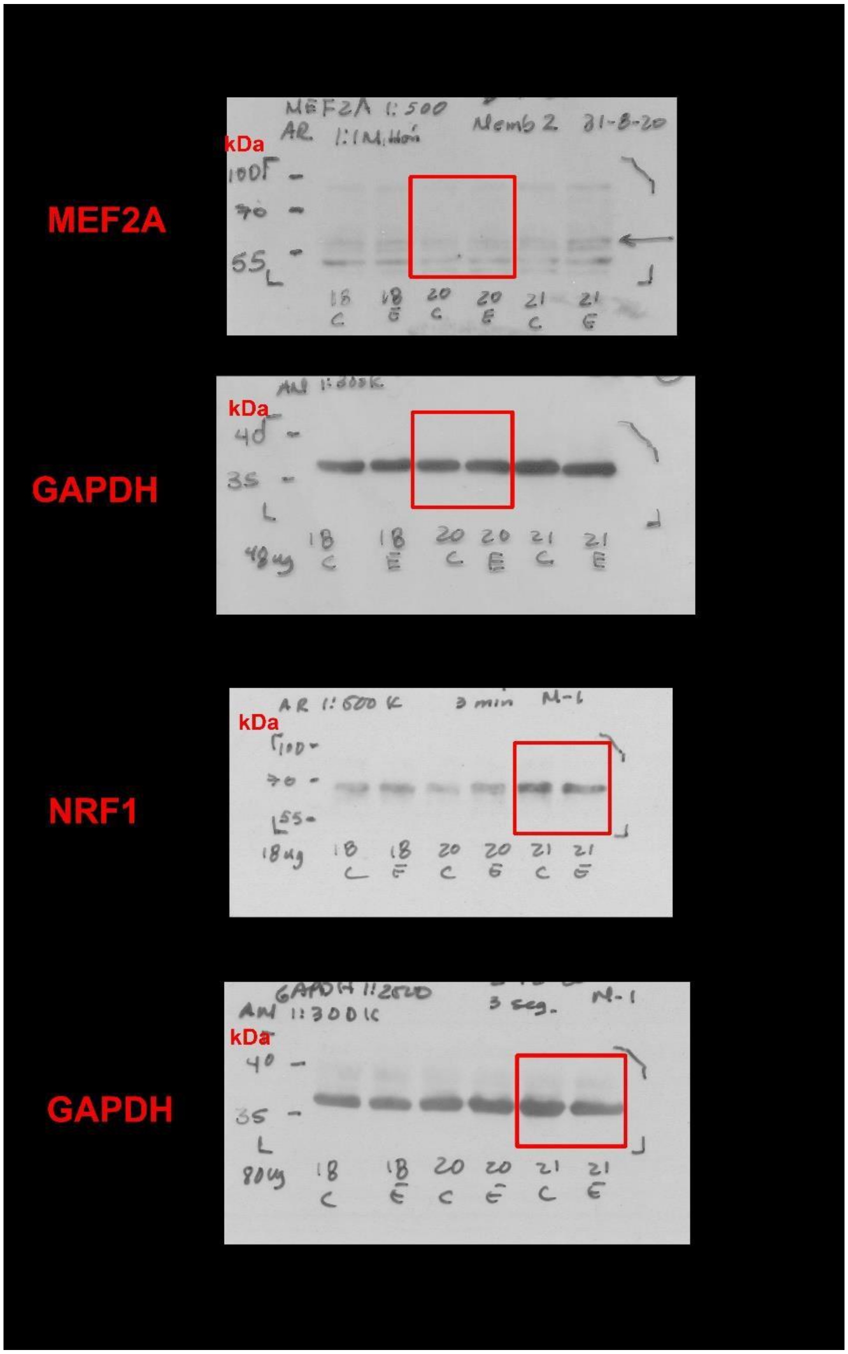

S13

Western blots showing the respective target bands of MEF2A and GAPDH from two separate experiments. H9c2 cells were transfected with a control plasmid (left lanes) or with a plasmid containing pre miR-223-5p (right lanes). Red boxes indicate areas of blots not shown in Figure 4**c**.

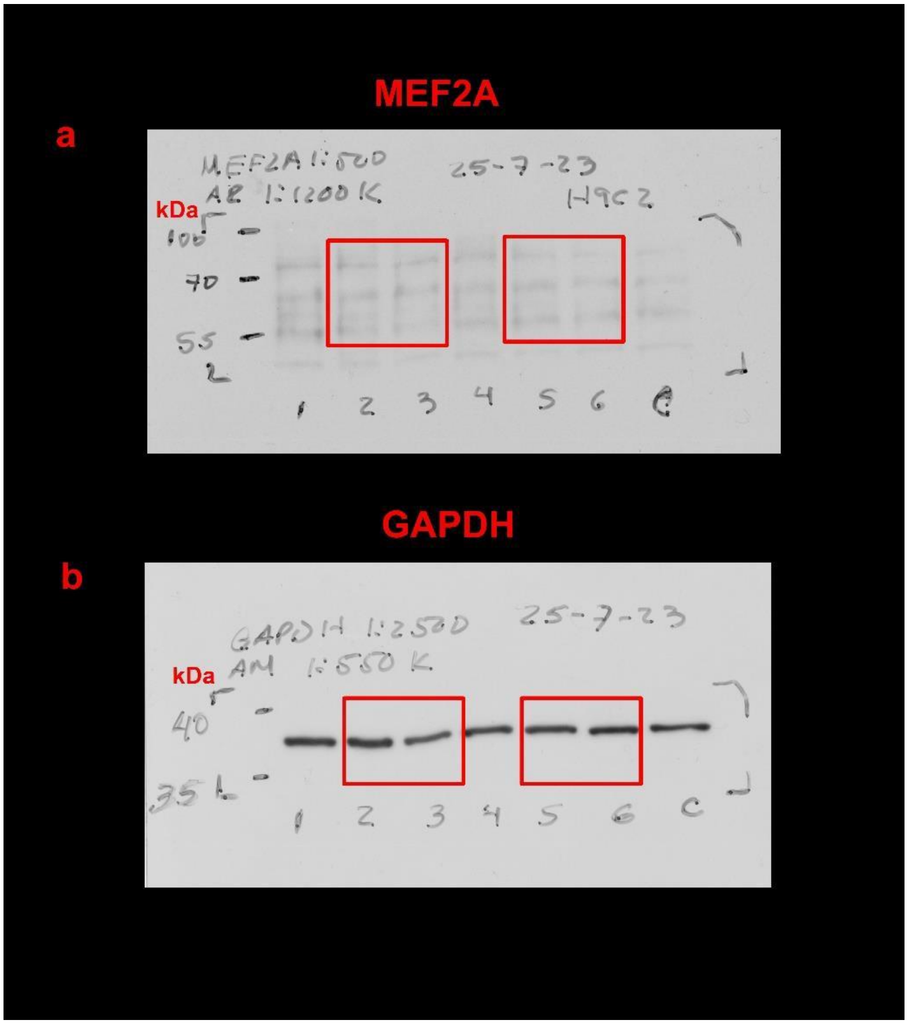

S14

Western blots showing the respective target bands of FOXO1 (**a** and **c**) and GAPDH (**b** and **d**) from two independent experiments.

Left lanes, control and right lanes, post exercise.

Red boxes indicate areas of blots not shown in Figure 5**b**.

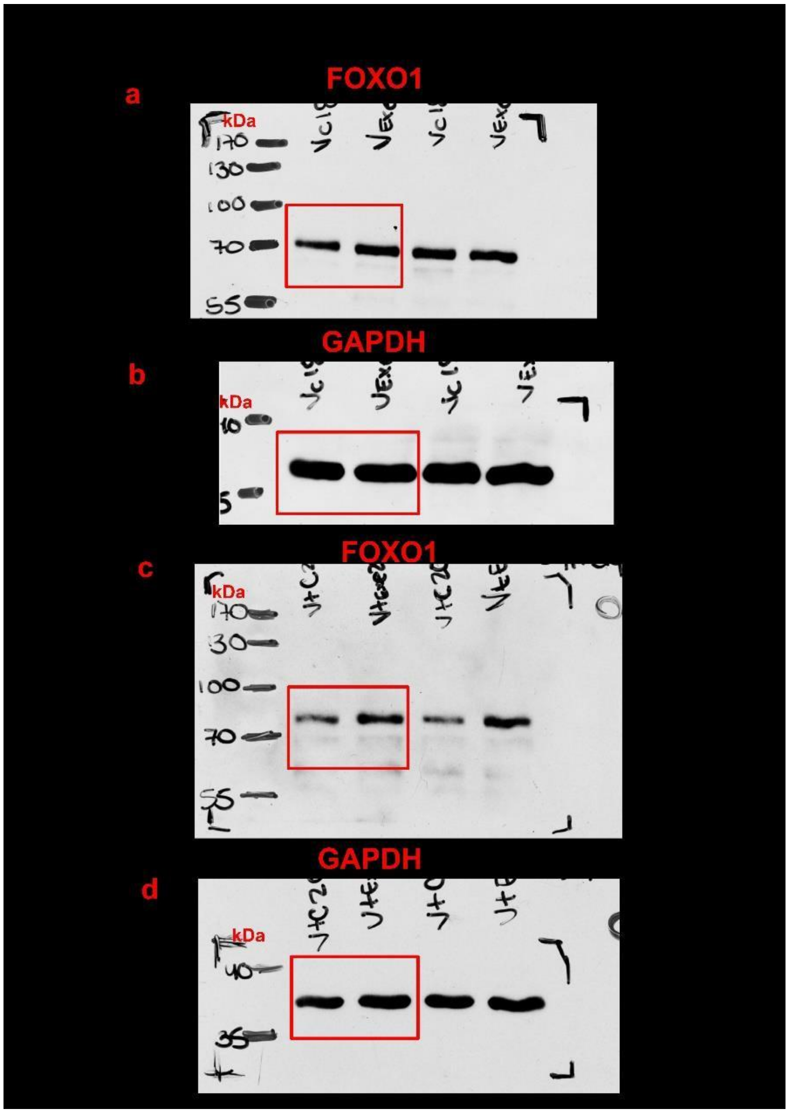

S15

Western blots showing the respective target bands of FOXO1 and GAPDH from three independent experiments (**a**-**c**).

Left lanes, control, and right lanes, 18 h post exercise.

Red boxes indicate areas of blots not shown in Figure 5.

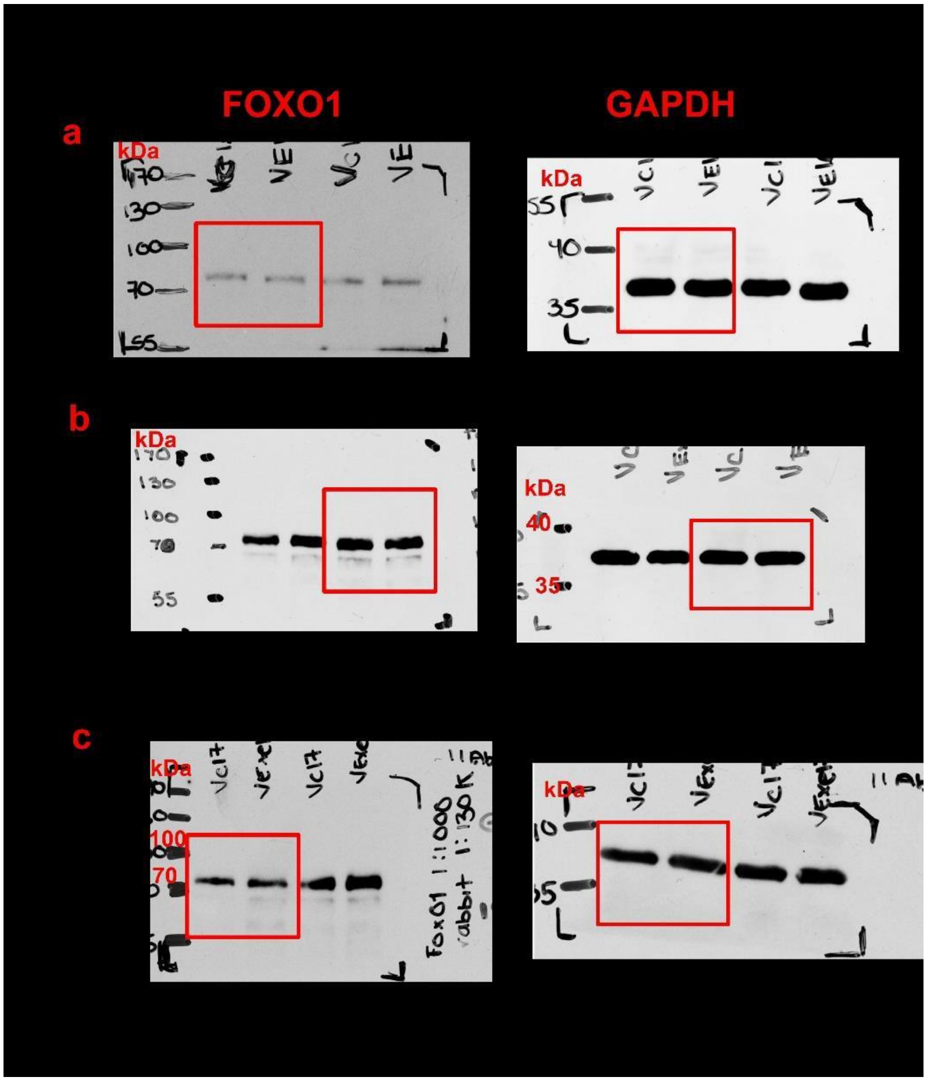

Supplement: Supplementary file 1 — Supplementary Figures. [file 41598_2023_41696_MOESM1_ESM.pdf]
